# Electronegative Low-Density Lipoprotein L5 Impairs Viability and NGF-Induced Neuronal Differentiation of PC12 Cells via LOX-1

**DOI:** 10.3390/ijms18081744

**Published:** 2017-08-11

**Authors:** Jiz-Yuh Wang, Chiou-Lian Lai, Ching-Tien Lee, Chen-Yen Lin

**Affiliations:** 1 Graduate Institute of Medicine, College of Medicine, Kaohsiung Medical University, Kaohsiung 80708, Taiwan; k00511882@gmail.com; 2 Department of Medical Research, Kaohsiung Medical University Hospital, Kaohsiung 80708, Taiwan; 3 Department of Neurology, Faculty of Medicine, College of Medicine, Kaohsiung Medical University, Kaohsiung 80708, Taiwan; cllai@kmu.edu.tw; 4 Department of Neurology, Kaohsiung Medical University Hospital, Kaohsiung 80756, Taiwan; 5 Department of Nursing, Hsin-Sheng College of Medical Care and Management, Taoyuan 32544, Taiwan; chingtien1213@gmail.com

**Keywords:** electronegative LDL, lectin-like oxidized LDL receptor-1 (LOX-1), nerve growth factor (NGF), neuronal differentiation, apoptosis

## Abstract

There have been striking associations of cardiovascular diseases (e.g., atherosclerosis) and hypercholesterolemia with increased risk of neurodegeneration including Alzheimer’s disease (AD). Low-density lipoprotein (LDL), a cardiovascular risk factor, plays a crucial role in AD pathogenesis; further, L5, a human plasma LDL fraction with high electronegativity, may be a factor contributing to AD-type dementia. Although L5 contributing to atherosclerosis progression has been studied, its role in inducing neurodegeneration remains unclear. Here, PC12 cell culture was used for treatments with human LDLs (L1, L5, or oxLDL), and subsequently cell viability and nerve growth factor (NGF)-induced neuronal differentiation were assessed. We identified L5 as a neurotoxic LDL, as demonstrated by decreased cell viability in a time- and concentration-dependent manner. Contrarily, L1 had no such effect. L5 caused cell damage by inducing ATM/H2AX-associated DNA breakage as well as by activating apoptosis via lectin-like oxidized LDL receptor-1 (LOX-1) signaling to p53 and ensuring cleavage of caspase-3. Additionally, sublethal L5 long-termly inhibited neurite outgrowth in NGF-treated PC12 cells, as evidenced by downregulation of early growth response factor-1 and neurofilament-M. This inhibitory effect was mediated via an interaction between L5 and LOX-1 to suppress NGF-induced activation of PI3k/Akt cascade, but not NGF receptor TrkA and downstream MAPK pathways. Together, our data suggest that L5 creates a neurotoxic stress via LOX-1 in PC12 cells, thereby leading to impairment of viability and NGF-induced differentiation. Atherogenic L5 likely contributes to neurodegenerative disorders.

## 1. Introduction

Dementia is the most common neurodegenerative disorder afflicting the aged. Growing evidence suggests a strong and likely causal association of cardiovascular disease (CVD) with cognitive decline and Alzheimer’s disease (AD) [1]. Actually, a high prevailing rate of often neglected cardiovascular problems is found in the AD population [1,2]. Epidemiological studies have established a strong relation between CVD and AD, but it remains unclear whether this relation is due to shared risk factors that independently influence disease progression, or if certain CVD risk factors contribute to AD pathogenesis by inducing neuronal damage or promoting the accumulation of plaques and tangles. Among CVD risk factors, elevated low-density lipoprotein (LDL) is reported to reduce cerebral perfusion, increase oxidative stress, and activate neuroinflammatory responses, all of which promote AD progression [3,4]. An association of high LDL with cognitive impairment and AD has been demonstrated; however, how plasma LDL induces dementia has yet to be clearly defined.

Hypercholesterolemia has been emphasized a risk factor for the development of AD [5,6]. Cholesterol is transported inside LDL particles in circulation, so high LDL-cholesterol (LDL-C) level may increase the individuals suffering from cognitive decline and dementia. Cholesterol-related studies suggest that there is a negative correlation between plasma LDL-C level and cognition [7,8], and that high LDL-C level is associated with faster cognitive degeneration in AD patients [9]. Moreover, LDL particles are known to cause mischief to both cardiovasculature and cerebrovasculature if they become oxidized and invade the endothelium. Oxidized LDL (oxLDL) can promote AD progression through inducing cerebrovascular dysfunction [10,11]. Thus, lowering LDL-C with statins has been an excellent strategy for reducing dementia risk [12].

One plausible mechanisms by which LDLs contribute to cognitive dysfunction and dementia progression is through the modification to electronegative LDL [LDL(−)], a class of naturally occurring atherogenic lipoproteins [13]. Similar to oxLDL, plasma LDL(−) is increased in patients with high CVD risk, including patients with hyperlipidemia, diabetes, severe renal disease, and nonalcoholic steatohepatitis [14]. Of note, L5 has the relatively highest electronegative charge among chromatographically resolved human LDL subfractions (L1–L5), and evidence has shown that L5 induces marked atherogenic changes and apoptosis in cultured vascular endothelial cells (ECs) [15]. Further, circulating L5 is moderately elevated in asymptomatic individuals with increased CVD risk, such as those with metabolic syndrome, hypercholesterolaemia and type 2 diabetes [16,17]. Nevertheless, to our knowledge, the role of L5 in neurodegenerative pathogenesis remains uncharacterized. Specifically, it remains uncertain whether L5 leakage into brain tissue under the situations of aberrant cerebrovasculature caused by cerebral amyloid angiopathy or increased blood-brain barrier (BBB) permeability induced by CVD-related vascular factors can directly induce neuronal dysfunction or death.

We hypothesized that L5 is neurotoxic, thereby leading to neurodegeneration. Thus, our purpose in this in vitro study was to examine whether L5 negatively impacts on neuron-like PC12 cell reactivities, such as cell viability and nerve growth factor (NGF)-induced differentiation. The related signaling cascades that L5 disturbs were assessed after the desired treatments. We found that L5 induces ataxia-telangiectasia mutated (ATM) protein/H2AX-associated DNA damage, causes cell apoptosis via lectin-like oxidized LDL receptor-1 (LOX-1)/p53/caspase-3, and inhibits neurite outgrowth by interacting with LOX-1 and reducing NGF-stimulated Akt activation. Here, we present novel findings showing that L5 induces neurotoxicity directly, thereby identifying elevated plasma L5 as a possible causal link between hypercholesterolemia-related CVD and neurodegeneration, such as AD.

## 2. Results

### 2.1. L5 Shows a Cytotoxic Property and Reduces the Viability of PC12 Cells

To characterize the action of L5 on neuronal cell reactivity, both cell viability and NGF-induced neuronal differentiation were assessed in cultured neuron-like PC12 cells. Two controls were included in most experiments, L1 as a negative control and oxLDL as a positive control. Both L5 and L1 are naturally occurring lipoproteins in human circulation; in contrast to L5, L1 possesses the lowest electronegative charge among fractions and is regarded as non-cytotoxic [15,18]. OxLDL is a known toxic lipoprotein that plays a pivotal role in the progression of atheromatous plaque via mechanisms, including the destruction of the arterial wall and induction of vascular cell apoptosis [19,20].

**Figure 1:**
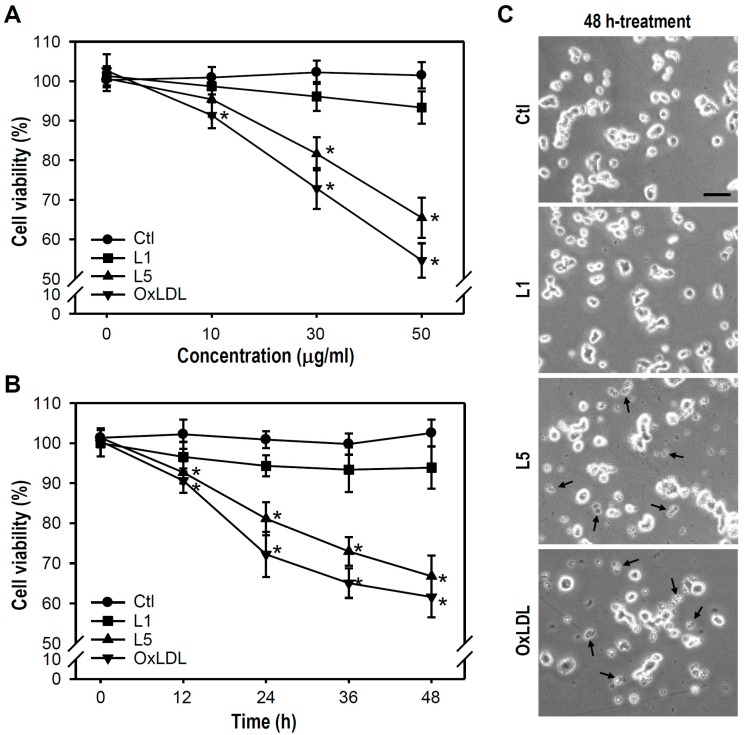
LDL fraction L5 shows a concentration- and time-dependent cytotoxicity to cultured PC12 cells. (**A**) Concentration-response relationships for L1 (negative control), L5, and oxLDL (positive control) were established. Indicated LDLs were applied for 24 h at various concentrations (0 to 50 μg/mL); (**B**) Time course was determined for L1, L5, and oxLDL cytotoxicity. Cultures were treated with the indicated LDL at 30 μg/mL for different times (0 to 48 h). In (**A**,**B**), the MTT reduction assay was applied to measure PC12 cell survival (viability) with untreated cells used as the control (Ctl). The number of live cells per well was calculated as a percentage of untreated control cell number. Each point is presented as the mean ± SEM from at least four independent experiments performed in triplicate. * *p* < 0.05 vs. Ctl at equal concentration (**A**) or at the same time point (**B**); and (**C**) Representative photos show an untreated culture and cultures treated with 30 μg/mL of the indicated LDL for 48 h. Fragmented cell debris (putative apoptotic bodies) are indicated by arrows. Scale bar = 50 μm.

We first examined whether L5 decreases the survival of PC12 cells by treating cultures with 0 to 50 μg/mL L1, L5, or oxLDL for 24 h, followed by MTT reduction assays (Figure 1A). Compared to the untreated control, L5 ≥ 30 μg/mL and oxLDL ≥ 10 μg/mL significantly reduced cell viability, while no such effects were observed in L1-treated cultures. Thus, we defined 30 μg/mL L5 as the threshold lethal concentration for PC12 cells. Next, cultures were exposed to 30 μg/mL of each LDL for times ranging from 0 to 48 h. Both L5 and oxLDL induced measurable cell death at 12 h and progressive cytotoxicity thereafter. In contrast, L1 entirely exerted no damaged effect on cell viability even after a 48 h exposure (Figure 1B). Regarding cell morphological changes in response to LDLs, a large amount of fragmented cell debris was detectable in L5- or oxLDL-treated cultures, but not in L1-treated cultures (Figure 1C). These results indicate that L5 induces both concentration- and time-dependent PC12 cell death.

### 2.2. L5 Induces Genotoxicity via ATM/H2AX Activation in PC12 Cells

**Figure 2:**
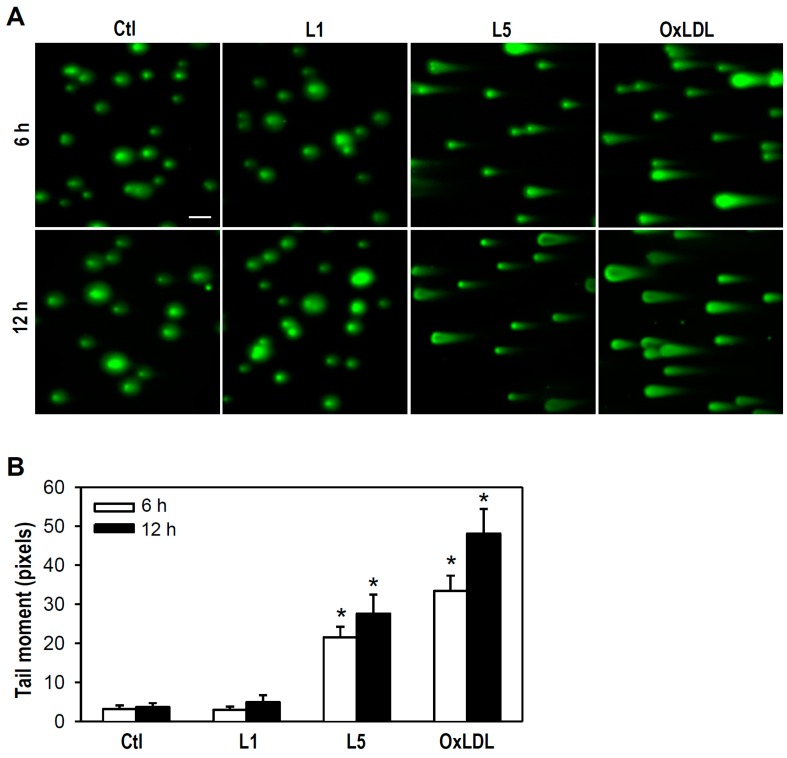
L5 induces DNA double-strand breaks (DSBs) in cultured PC12 cells. (**A**) Cultures treated with the indicated LDL (30 μg/mL) for 6 or 12 h were analyzed for DSBs using a neutral comet assay kit. Untreated cells were used as a control. Representative images from one of four independent experiments are presented. Scale bar = 50 μm; and (**B**) The comet tail moment used to determine DNA damage was quantified by COMETscore.v1.5 software. Data are presented as mean ± SEM from four independent experiments. * *p* < 0.05 vs. Ctl at the same time point. At least 200 cells were analyzed in each treatment group per experiment.

Next, we assessed whether L5-induced cell death involves DNA damage, such as the formation of double-strand breaks (DSBs) in chromatin, using a neutral comet assay. Under the experimental conditions tested in our study, both L5 and oxLDL, but not L1, caused DSBs as evidenced by the appearance of migrating DNA fragments (reflected by the length of the comet tail) (Figure 2A). Further, compared to control cultures, clear comet tails were observed in cultures treated with L5 or oxLDL at 6 and 12 h (Figure 2B), indicating that L5 has a genotoxic property that leads to destruction of DNA structure.

**Figure 3:**
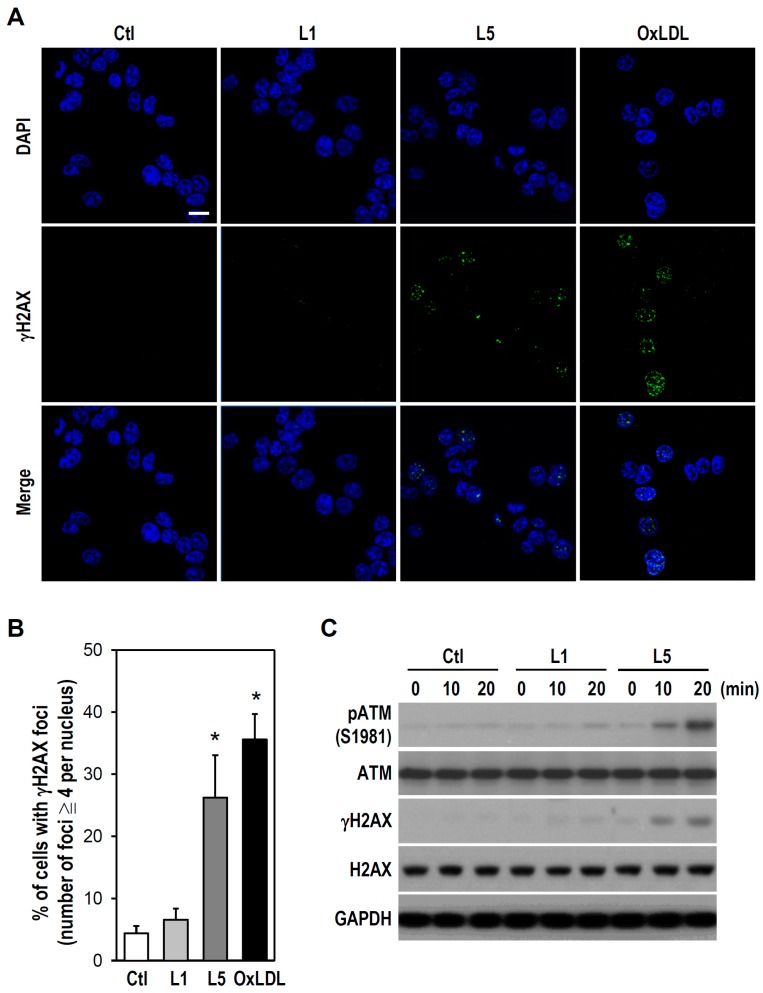
L5 induces ATM phosphorylation and formation of γH2AX foci in cultured PC12 cells. (**A**) Representative immunofluorescence images of γH2AX foci are shown. Cells grown on coverslips were treated with the indicated LDL (30 μg/mL) for 30 min. Untreated cells were used as a control. Immunostaining was performed using an antibody against γH2AX (green). Cells were counterstained with DAPI to recognize the nucleus (blue). Scale bar = 10 μm; (**B**) The histogram shows the quantification of (**A**). At least 20 randomly selected fields for each treatment group were examined. Cells with ≥ 4γH2AX foci per nucleus were regarded as positive and then scored. Value expressed as a percentage by calculating the number of positive cells (% of total). Data are presented as mean ± SEM from four independent experiments. * *p* < 0.05 vs. Ctl; and (**C**) After treating the cultures with L1 or L5 at 30 μg/mL for different times as indicated, western blotting was performed to detect pATM, ATM, γH2AX, and H2AX expression. GAPDH was a protein loading control. Representative blots selected from one of four independent experiments are presented.

Studies have suggested that DSBs formation is closely correlated with activation of both ATM and H2AX [21]. Hence, we examined whether DSBs induction by L5 is associated with ATM/H2AX activation. Immunostaining revealed the formation of γH2AX (H2AX phosphorylation on serine 139), which positively corresponds to DNA DSBs. Both L5 and oxLDL induced γH2AX foci formation in the nucleus as evidenced by punctate immunofluorescence signals, while such γH2AX immunoreactivity was not observed in untreated control or L1-treated cultures (Figure 3A,B). To further verify L5-induced genotoxicity, ATM and H2AX activation were determined by western blotting. Consistent with immunostaining results, a rapid and marked increase in both pATM and γH2AX was observed after only 10 min of L5 treatment, but not L1 treatment (Figure 3C). Together with the data presented in Figure 2, these results suggest that L5 injures PC12 cells by inducing ATM/H2AX-associated DNA DSBs.

### 2.3. L5 Induces Apoptotic Death of PC12 Cells via the LOX-1/p53/Caspase-3 Pathway

**Figure 4:**
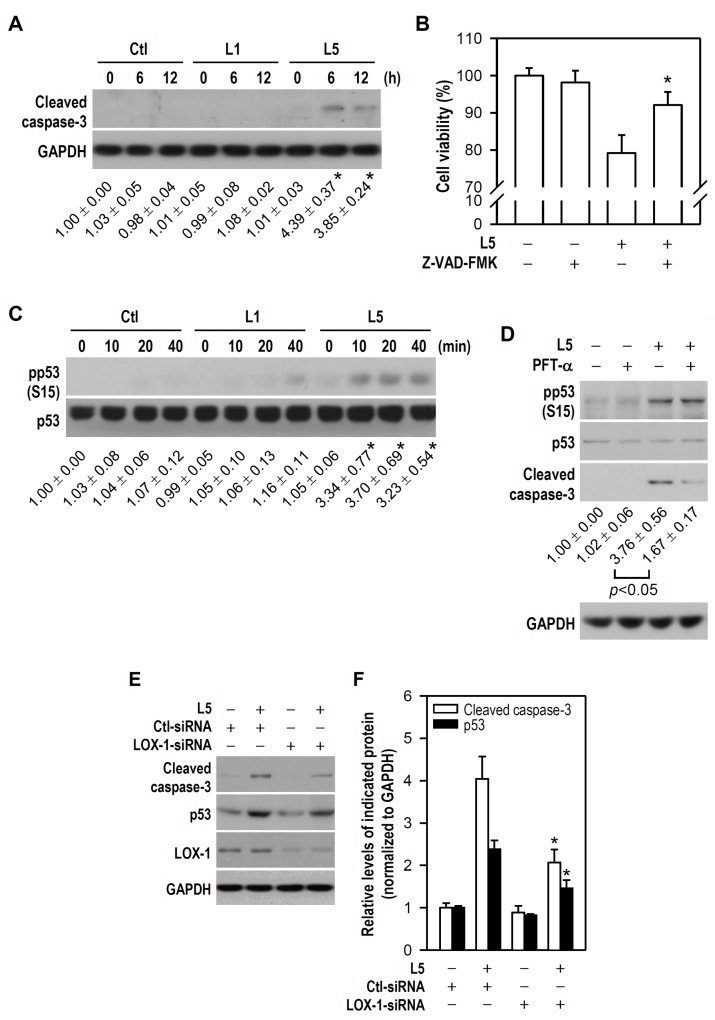
Reduced viability of L5-treated PC12 cells is associated with LOX-1 expression, p53 activation, and caspase-3 cleavage. (**A**) Cultures were treated with L1 or L5 (30 μg/mL) for the indicated times and then western blotting was used to detect cleaved caspase-3. Untreated cells were used as a control. GAPDH was a protein loading control. Quantitative values are expressed relative to untreated control at a time point of 0 h (assigned a value of 1); the means ± SEM from four independent experiments are shown below the representative blots. * *p* < 0.05 vs. Ctl at the same time point; (**B**) Cells were treated with or without L5 (30 µg/mL) for 12 h in the absence or presence of a pan-caspase inhibitor Z-VAD-FMK (20 μM). The histogram shows the cell viability of each treatment group relative to the untreated control (assigned a value of 100%). Data are presented as mean ± SEM from at least three independent experiments performed in triplicate. * *p* < 0.05 vs. L5 alone; (**C**) After the indicated treatments, western blotting was used to detect pp53 and p53 expression. Quantitative results of relative pp53 levels from four independent experiments are shown below the representative blots. * *p* < 0.05 vs. Ctl at the same time point; (**D**) Before sham or L5 (30 μg/mL) treatment for either 20 min or 12 h, cells were pretreated with or without PFT-α (10 μM) for 1 h. The expression of pp53 and p53 (at 20 min), and cleaved caspase-3 (at 12 h) was detected by western blotting. Relative quantitative results of cleaved caspase-3 from four independent experiments are shown below the representative blots; (**E**) One day after transfection with a scrambled siRNA (Ctl-siRNA) or a LOX-1-siRNA, cultures were treated with or without L5 for 12 h. Cleaved caspase-3, p53, and LOX-1 were detected by western blotting and representative blots are presented; and (**F**) Relative quantitative expression of cleaved caspase-3 and p53 in (**E**) is shown by a histogram. Data are from four independent experiments. * *p* < 0.05 vs. Ctl-siRNA + L5.

Damage to genomic integrity is known to cause apoptosis [22,23], and indeed many of the debris fragments observed following L5 or oxLDL treatment resembled apoptotic bodies (Figure 1C). Accordingly, we examined whether L5 causes apoptotic death in PC12 cells and activates apoptosis-associated signal transduction pathways. Compared to control, L5 increased cleaved caspase-3 (a key apoptotic caspase) expression after 6 or 12 h of treatment while L1 had no such effect (Figure 4A); moreover, this apoptotic insult was evidently inhibited by the pan-caspase inhibitor Z-VAD-FMK (Figure 4B). Subsequently, p53 activity was examined due to its close association with apoptosis and activation upon cellular stresses such as DNA damage. Furthermore, p53 is a direct substrate of ATM [24]. ATM rapidly phosphorylates p53 at Ser15 during early phase of DNA damage response, thereby reducing the interaction of p53 with MDM2, a negative p53 regulator that inhibits accumulation of p53 by targeting it for ubiquitination and proteasomal degradation [25,26]. Compared to the control, L5-treated cultures (but not L1-treated cultures) exhibited phosphorylated p53 elevation after as little as 10 min of exposure (Figure 4C). Moreover, the p53 inhibitor pifithrin-α (PFT-α) electively reduced the cleaved caspase-3 level in L5-treated cultures without altering the L5-phosphorylated p53 level, indicating that p53-dependent apoptosis is a major mediator of L5-induced PC12 cell death (Figure 4D).

To identify the upstream factors leading to activation of the p53/caspase-3 pathway, we first focused on possible receptors involved. It has been shown that L5 signals and is internalized through LOX-1, and that the binding complex of L5/LOX-1 plays a critical role in L5-induced apoptosis of vascular ECs [15]. Accordingly, we examined whether LOX-1 participates in L5-induced induction of p53/caspase-3-dependent apoptosis in PC12 cells. Compared to L5-treated cultures transfected with control small interfering RNA (Ctl-siRNA), silencing of endogenous LOX-1 by RNA interference using a LOX-1-siRNA not only downregulated LOX-1 protein expression, as determined by western blotting, but also markedly reduced both cleaved caspase-3 level and p53 accumulation in response to L5 (Figure 4E,F). Noteworthily, this reduced p53 accumulation might be primarily because LOX-1-siRNA disturbs L5-induced p53 phosphorylation and then MDM2-mediated p53 degradation occurs. This finding suggests that LOX-1 is required for L5-induced downstream activation of p53/caspase-3. Taken together, these results strongly suggest that LOX-1/p53/caspase-3 signaling mediates L5-induced PC12 cell apoptosis.

### 2.4. L5 Inhibits NGF-Induced Differentiation of PC12 Cells

Neurite outgrowth in PC12 cells in response to NGF stimulation is a widely used model to examine neuronal differentiation and development. In addition to assessing L5 effects on PC12 cell viability, we further examined whether L5 disturbs NGF-induced neuronal differentiation of PC12 cells. According to above results indicating that L5 < 30 μg/mL did not cause detectable PC12 cell death (Figure 1A), we defined 10 μg/mL as the sublytic concentration to assess the impact of L5 on PC12 cell differentiation induced by NGF.

**Figure 5:**
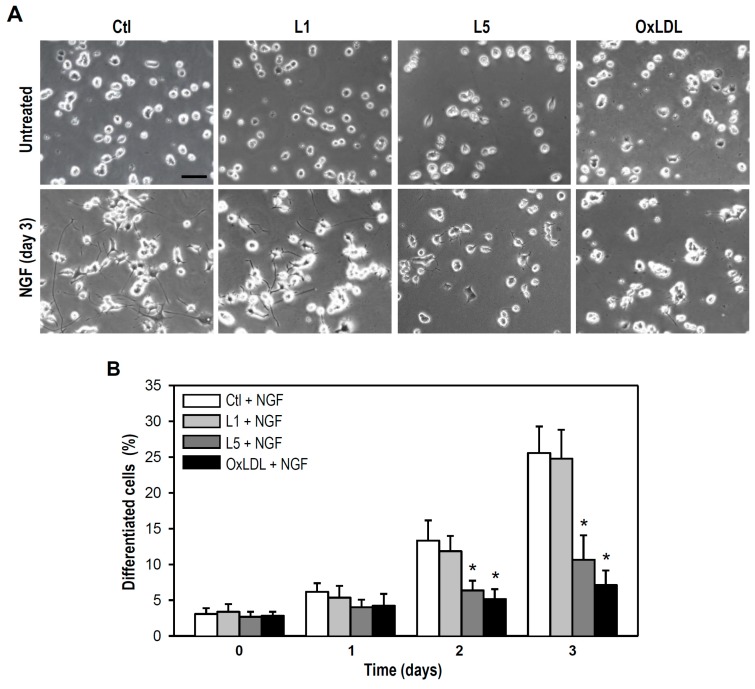
L5 inhibits NGF-induced neuronal differentiation of cultured PC12 cells. (**A**) Representative photos show PC12 cells treated with the indicated LDLs (10 μg/mL) in the absence or presence of NGF (50 ng/mL) for 3 days. Cells untreated with LDLs were used as a control. NGF-induced neuronal differentiation is indicated by the extent of neurite outgrowth. Scale bar = 50 μm; and (**B**) The percentage of neuronal differentiation in PC12 cells exposed to LDLs (10 μg/mL) plus NGF (50 ng/mL) for 1, 2, and 3 days was determined by calculating the number of cells (% of total) bearing neurites longer than one cell body in diameter. Ten randomly selected fields were examined and at least 300 cells were counted for each treatment group at each time point. Data are presented as mean ± SEM of one representative experiment; two other experiments revealed consistent results. * *p* < 0.05 vs. Ctl + NGF at the same time point.

As shown in Figure 5A, both L5 and oxLDL interfered with neurite outgrowth in PC12 cultures subjected to long-term NGF exposure, as evidenced by generally shorter neurites compared to control NGF-treated cultures on day 3 of treatment. As expected, cultured cells treated with L1 plus NGF displayed normal outgrowth of neurites. This finding implies that L5 at sublytic concentration is capable of disturbing NGF-induced neurite outgrowth. Indeed, both L5 and oxLDL but not L1 markedly lowered the proportion cells showing NGF-induced morphological differentiation (defined as cells with neurites longer than one soma in length) on days 2 and 3 of treatment compared to control cultures incubated with NGF (Figure 5B).

**Figure 6:**
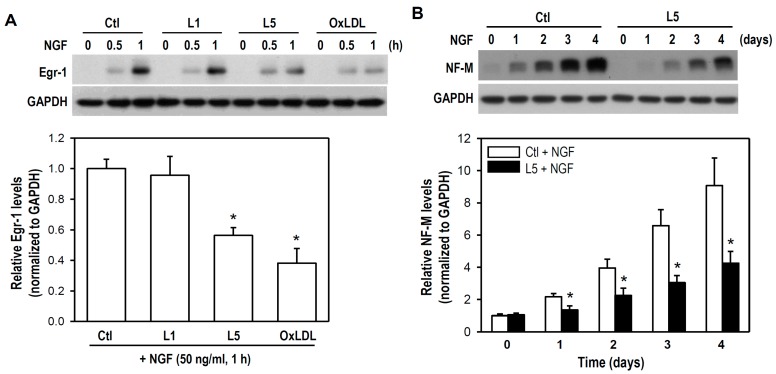
L5 inhibits NGF-induced upregulation of Egr-1 and NF-M. (**A**) Cultures were treated with or without the indicated LDLs (10 μg/mL) for 1 h, followed by NGF (50 ng/mL) addition for the indicated time periods. Cells untreated with LDLs were used as a control. Egr-1 expression was detected by western blotting (representative blot is shown). GAPDH was a protein loading control. The histogram shows relative Egr-1 expression values of each treatment group at a time point of 1 h. Data are presented as mean ± SEM from four independent experiments. * *p* < 0.05 vs. Ctl + NGF; and (**B**) Cultures were treated with L5 (10 μg/mL) for 1 h, followed by NGF (50 ng/mL) addition for another times (days) as indicated. Cells untreated with LDLs were used as a control. Western blotting was performed to detect NF-M expression (representative blot of NF-M is shown). The histogram shows relative NF-M levels between Ctl + NGF and L5 + NGF groups from day 0 to 4. Data are from four independent experiments. * *p* < 0.05 vs. Ctl + NGF on the same day.

Early growth response factor-1 (Egr-1) is a NGF-responsive transcription factor and exerts long-term effects on neural cell growth, while neurofilament is critical for radial axon growth and determines axon caliber [27,28]. Therefore, we further determined the expression levels of both Egr-1 and neurofilament-medium (NF-M) under the conditions of NGF combined with distinct LDLs. The expression of Egr-1 was induced within 1 h after NGF addition, and this response was markedly reduced by both L5 and oxLDL but not by L1 (Figure 6A). Further, NGF induced an elevation in neurite growth-associated NF-M expression level, and again this response was evidently attenuated by L5 over a 4-day experimental period (Figure 6B). Thus, L5 appears to have a negative impact on neurodifferentiation-associated upregulation of Egr-1 and NF-M. Collectively, these data suggest that a sublytic concentration of L5 (10 μg/mL) remains still neurotoxic and impairs neuronal development by inhibiting NGF-induced differentiation.

### 2.5. L5 Weakens NGF-Induced Akt but Not TrkA and MAPKs Activation

**Figure 7:**
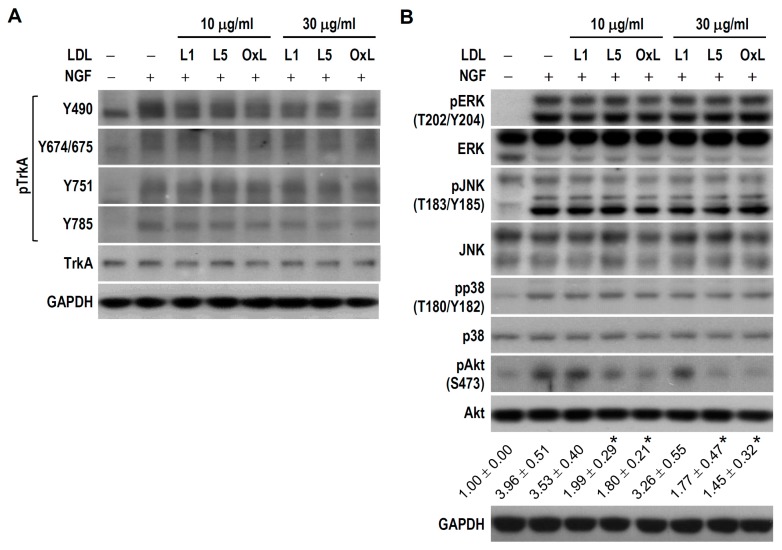
L5 decreases the level of phospho-Akt (pAkt), but not pTrkA and pMAPKs, in NGF-treated PC12 cells. (**A**) Cultures were treated with L1, L5, or oxLDL (OxL) at 10 or 30 μg/mL for 1 h, followed by NGF (50 ng/mL) addition for another 20 min. Untreated cells were used as a control. Western blotting was used to detect the expression of TrkA phosphorylated at various tyrosine sites and total TrkA protein. GAPDH was a protein loading control. Representative blots selected from one of four independent experiments are presented; and (**B**) Alternatively, western blotting was performed to detect pERK, ERK, pJNK, JNK, pp38, p38, pAkt, and Akt expression. Quantitative values of pAkt relative to the level of untreated control (assigned a value of 1) are expressed; the means ± SEM from four independent experiments are shown below the representative blots. * *p* < 0.05 vs. NGF alone.

We then examined the upstream signaling pathways responsible for L5 suppression of NGF-induced differentiation in PC12 cells. Given that many NGF effects are mediated by activation of its receptor TrkA and downstream signaling kinases such as MAPKs and PI3K/AKT, we examined whether L5 weakens NGF-induced TrkA phosphorylation and resultant activation of MAPKs (ERK, JNK, and p38) and Akt in PC12 cultures. We first measured the phosphorylation status of several crucial residues within the tyrosine kinase domain of TrkA because autophosphorylation at these sites reflects TrkA activity or is required for activation of downstream kinase cascades [29]. In response to NGF stimulation, all tested tyrosine residues within TrkA including Tyr490, Tyr674/675, Tyr751, and Tyr785 were rapidly phosphorylated; however, no obvious difference in phosphorylation was found at any one of these tyrosine sites between cultures treated with NGF alone versus NGF plus L1, L5, or oxLDL (Figure 7A). This indicates that L5 has no influence on TrkA catalytic activity or on TrkA coupling to downstream Ras/MAPK, PI3K/Akt, and phospholipase Cγ. Further, no detectable difference was observed in the phosphorylation levels of ERK, JNK, and p38 between cultures treated with NGF alone and those treated with NGF plus L1, L5, or oxLDL. In contrast, NGF-induced Akt phosphorylation was markedly reduced in L5- and oxLDL-treated cultures at both 10 and 30 μg/mL compared to cultures treated with NGF alone, while such a repressive effect was not found in cultures incubated with L1 plus NGF (Figure 7B). These results suggest that L5 represses NGF-induced neuronal differentiation in PC12 cells by interfering with the TrkA/PI3K/Akt pathway.

### 2.6. LOX-1 Is Required for L5 Suppression of NGF-Induced Akt Phosphorylation

**Figure 8:**
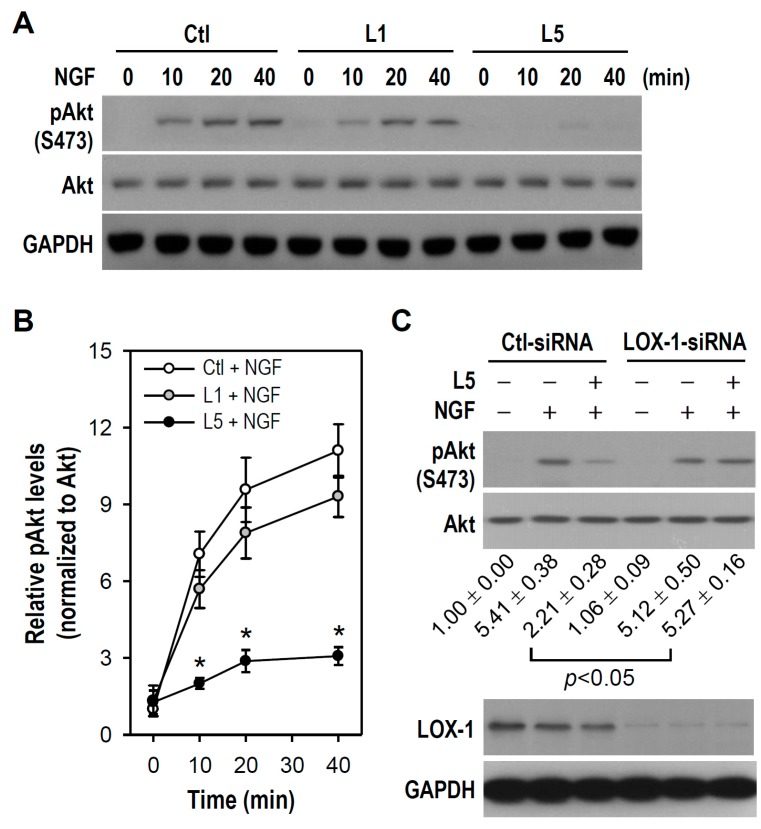
LOX-1 knockdown abolishes L5-induced Akt phosphorylation decline in NGF-treated PC12 cells. (**A**) Cultures were treated with L1 or L5 (10 μg/mL) for 1 h, followed by NGF (50 ng/mL) addition for the indicated time periods. Cells untreated with LDLs were used as a control. Western blotting was used to detect pAkt and Akt expression. GAPDH was a protein loading control. Representative blots selected from one of four independent experiments are presented; (**B**) According to the results in (**A**), quantitative analyses of relative pAkt levels were done in each treatment group. Data are presented as the mean ± SEM from four independent experiments. * *p* < 0.05 vs. Ctl + NGF at the same time point; and (**C**) One day after transfection with siRNA as indicated, cultures were treated with or without L5 (10 μg/mL) for 1 h and incubated in the absence or presence of NGF (50 ng/mL) for another 20 min. Western blotting was conducted to detect pAkt, Akt, and LOX-1 expression. Quantitative results of relative pAkt levels were obtained from four independent experiments and are shown below the representative blots.

To verify abovementioned findings that NGF-induced Akt activation is reduced in L5-treated cultures, cultures were treated with NGF alone or NGF combined with L1 or L5 for brief periods and Akt phosphorylation/activation status was measured. As shown in Figure 8A, phosphorylated Akt became detectable within 10–40 min after the onset of NGF treatment, and this response was reduced in the presence of L5 but not L1. The suppressive effect of L5 on NGF-induced Akt phosphorylation reached significance at time points of 10, 20, and 40 min compared to control group treated with NGF (Ctl + NGF) (Figure 8B).

Results present in Figure 4 strongly suggest that LOX-1 is involved in PC12 cell apoptosis induced by L5. We also explored the possibility that LOX-1 participates in L5-induced suppression of neuronal differentiation in NGF-treated PC12 cells. Because NGF-stimulated Akt activation is impaired by L5, we examined if L5 weakens the TrkA/PI3K/Akt signal cascade via LOX-1. As shown in Figure 8C, NGF-induced stimulation of Akt phosphorylation was not reduced by L5 in cultures transfected with a LOX-1-siRNA, but was weakened by L5 in cultures transfected with a Ctl-siRNA. Therefore, these results suggest that L5 attenuates NGF-activated Akt via LOX-1, and that LOX-1 acts as a mediator coupling extrinsic L5 to intracellular PI3K/Akt pathway.

## 3. Discussion

Frequent comorbidity of AD with CVD suggests that vascular problems contribute to cognitive decline and AD-associated dementia. High circulating LDL levels, a strong CVD risk factor, are associated with increased production of cerebrovascular amyloid β (Aβ) peptide and increased risk of AD [4]. Further, more studies also suggest that elevated blood cholesterol and triacylglycerol increase the likelihood of AD [6]. Cerebrovasculature damage induced by circulating LDL or other vascular mechanisms may enhance access of detrimental LDLs to brain parenchyma, resulting in neurotoxicity. Indeed, this study suggests that in a situation of BBB breach, L5 may directly impair neuronal viability and NGF effects, such as neurodifferentiation. Thus, it is a feasible strategy for prevention of AD and other forms of age-related cognitive impairment by aggressively targeting hypercholesterolemia.

Numerous studies have highlighted a negative role of L5 in the progression of CVDs, especially atherosclerosis, and thus L5 is regarded as a potent atherogenic LDL. It has been indicated that L5 is abundant in dyslipidemic but not normolipidemic human plasma, and hyperlipidemia is also a shared factor for AD [3]. Shen et al. found a functional link between pro-atherogenic lipoproteins and platelet-mediated thrombus formation in stroke, and pointed out that L5 is substantially elevated (40-fold higher than control subjects) in ischemic stroke patients [30]. Moreover, L5 can cause Aβ peptide secretion from platelets, which contributes to platelet hyper-reactivity and stroke complications [31]. These clinical results suggest the possibility of L5 directly impacting neurological functions, such as cognition, under the premise of impaired BBB. In the present work, we identify a negative role of L5 in basal viability and NGF-induced neuronal differentiation of PC12 cells, consistent with a neurotoxic effect in aging humans. Further investigations aimed at establishing the clinical relevance of plasma L5 to AD-related neurodegeneration are clearly warranted.

It is highly possible that plasma LDL penetrates the BBB due to a broken architecture of cerebral microvasculature during the progression of AD or CVD, which in turn leads to neuronal damage and neuroinflammation-related glial activation. In fact, apolipoprotein B (apoB) or LDL has been detected in cerebrospinal fluid (CSF) or/and central nervous system under pathological conditions. For example, apoB/LDL was found or obviously elevated in CSF collected from patients with subarachnoid hemorrhage, cerebrotendinous xanthomatosis, or tuberculous meningitis [32,33,34]. Plasma apoB was co-localized with cerebral Aβ in the plaques of transgenic AD mice as revealed by a 3-dimensional immunomicroscopy; further, plaque abundance in these mice positively correlated with apoB [35,36]. Moreover, BBB breakdown has been described in chronic CVDs, and peripheral inflammatory response is a cause of enhanced BBB permeability [37]. CVDs like arteriosclerosis and hypertension are characterized by increased production of pro-inflammatory cytokines in blood [38], and administration of pro-inflammatory cytokines such as TNF-α, IL-1β, and IL-6 to monolayers of cerebral ECs or pericyte/endothelial cell co-cultures leads to BBB disruption [39,40]. Clinical studies show that diseases compromising the vascular system, such as hypertension, hypercholesterolemia, and diabetes, are able to disrupt BBB integrity and increase the risk of AD [41]. Autopsy studies have also shown that vascular abnormalities, including endothelial damage-associated microangiopathy, were universally observed in AD brain tissue [42]. Together, these studies revealing the occurrence of BBB dysfunction in CVDs or encephalopathies set a stage for this study and suggest that L5 and other neurotoxic agents in plasma may directly access neurons via a damaged BBB.

Actually, L5 does not exist in healthy individuals with clinically normolipidemia; certainly, L5 does not appear in the brain under normally physiological conditions. However, plasma L5 likely extravasates directly into brain milieu in certain pathological cases, such as BBB dysfunction. Moreover, the L5 concentration (10 or 30 μg/mL) used in this study should not be present in humans with normal or pathological CNS states, but is approximately equal to or less than other in vitro studies examining the impact of L5 or LDL(−) on the pathogenic process of atherosclerosis. For example, Chu et al. used 50 μg/mL of L5 for 24 h to examine C-reactive protein expression in vascular ECs [43]. Estruch et al. assessed cytokine release in monocytes treated with LDL(−) at 150 μg apoB/mL [44]. Because in vitro L5 toxicity has a tendency towards a concentration- and time-dependency, we can reasonably consider that neurotoxicity is attainable in vivo if using lower L5 concentrations (<10 μg/mL) for longer treatment periods (a few weeks or months) under the premise of impaired BBB. To elucidate this thought, a long-term exposure to low plasma L5 levels will be applied to future animal studies using transgenic AD mice, and hypertensive or hypercholesterolemic rodents.

It is worthily noticed that the differential effects of L1 and L5 on PC12 cell viability and NGF-induced neuronal differentiation are mainly associated with activation of different cell surface receptors and intracellular signaling pathways. Several properties ascribed to L5, such as highly electronegative charge, higher aggregation potential, conformationally distinct apoB, and greater content in inflammatory lipids, suggest that L5 interacts with different receptors than L1. Due to this higher electronegativity than L1, it was thought that L5 binds to the LDL receptor (LDLR) with less affinity. Avogaro et al. observed that the more electronegative LDL subfraction exhibited a lower binding capacity for LDLR [45]. This finding is similar to Benitez et al. who found that LDLR affinity was three-fold lower for LDL(−) than for native LDL [46]. The low binding affinity of L5 to LDLR may be partly explained by high nonesterified fatty acid content [46], increased degree of aggregation [47], and abnormal conformation of apoB [48]. The consequence of this loss of LDLR affinity is a diminished L5 clearance from human circulation, allowing for further deleterious effects on vascular cells and neurons.

Regarding cell surface receptor for L5 binding, Chen and coworkers reported that L5, a highly pure form of LDL(−), is a ligand for LOX-1, but not normal LDLR. L5 recognizes, signals through, and is internalized by LOX-1, which has high affinity for negatively charged ligands, whereas the less electronegative LDL subfractions L1–L4 rely on the LDLR for biological effects [15,18]. In addition to L5, LOX-1 is also activated by oxLDL, reactive oxygen species (ROS), endothelin-1, angiotensin II, advanced glycation end products, and shear stress. Previous studies have demonstrated activation of diverse downstream signaling pathways upon binding of L5 to LOX-1. For example, L5 suppresses Akt phosphorylation via LOX-1 to impair Akt-mediated growth and survival signals in bovine aortic ECs [15]. The L5/LOX-1 complex also triggers ROS production to induce oxidative stress in human aortic ECs [43], and stimulates overexpression of various adhesion molecules and inflammatory chemokines, thus promoting monocyte adherence to vascular endothelium, an early pathogenic event for atherosclerosis [49]. L5 binding to LOX-1 induces the apoptosis of human aortic ECs via p38 MAPK/caspase-3 signaling [50]. L5/LOX-1-activated platelets switch on a signaling pathway including IκB kinase 2 and nuclear factor-κB activation, which is critical for stroke pathobiology [31]. Further, we found that L5 signals to ATM/H2AX and p53/caspase-3 cascades through LOX-1, resulting in PC12 cell DNA breaks and apoptosis. Based on these findings, we conclude that LOX-1 transmits damage signals from L5 but not L1, thereby accounting for the unique cytotoxic effects of L5 in PC12 cells.

Once DNA damage such as DSBs is elicited by genotoxic insults like radiation and free radicals, apoptosis may be activated if this damage is not repaired [22]. We identified L5 as a genotoxic agent that injures PC12 cells through DNA DSBs and apoptotic death, and further found out the intermediary signaling pathways. Several lines of evidence suggest that ATM is activated in response to DNA damage, which then promotes H2AX phosphorylation at DSB sites (manifested as nuclear foci) for initiation of DSB repair [21,22]. As expected, γH2AX foci formation was observed by immunofluorescence, and increased phosphorylation levels of both ATM and H2AX were revealed by western blotting after L5 stimulation. In addition, activated p53 exerts pro-apoptotic effects by transcriptionally activating genes involved in cell cycle arrest, inhibition of proliferation, and induction of senescence, all of which are conducive to the maintenance of genomic integrity following genotoxic insults [51]. Indeed, we found that L5 rapidly triggers p53 phosphorylation accompanied by caspase-3 activation and PC12 cell apoptosis. This finding was further verified by the p53 inhibitor PFT-α, which effectively lowered the cleaved caspase-3 level and inhibited apoptosis.

In this study, L5-induced PC12 cell apoptosis also relied on LOX-1 expression, as evidenced by the reduction of L5-elevated caspase-3 cleavage and p53 accumulation levels in cells transfected with LOX-1 siRNA. These results concur with those of Li et al. who reported that LOX-1 is mainly found in cortical neurons, and its upregulation is involved in neuronal apoptosis [52]. To mediate L5 cytotoxicity, we speculate that unknown intracellular transmitters triggered by L5/LOX-1 complex should signal to ATM/H2AX and p53/caspase-3 cascades, followed by DNA breaks and apoptotic death in PC12 cells. Of note, L5 but not L1 has been indicated to rapidly elicit ROS production via LOX-1 and functions similarly to oxLDL in inducing oxidative stress [43]. Moreover, ROS is able to directly evoke p53-dependent apoptosis in PC12 cells [53], and the accumulation of ROS caused by polyQ proteins activates ATM/ATR-dependent DNA damage response [54]. ATM acts upstream of p53 in a signal transduction pathway initiated by ionizing radiation and can directly activate/phosphorylate p53 in vivo [24]. Thus, it is possible that ROS serves as a stress signal transmitter that connects the L5/LOX-1 complex to ATM/H2AX and p53/caspase-3 pathways in our PC12 cell model. Further work is required to verify this inference.

Morphological differentiation of neurons is essential for cell-cell communication and information processing. Differentiation begins with neurite sprouting, followed by progressive elongation of axons and elaboration of dendrites. Regarding this, we found that L5 impaired NGF-induced neurite outgrowth through weakening Akt phosphorylation. Actually, PI3K/Akt cascade has been suggested to be critical for NGF-induced PC12 cell differentiation [55,56]. Moreover, LOX-1 knockdown abolished L5-mediated inhibition of Akt activation by NGF, indicating that L5 requires interaction with LOX-1 to inhibit NGF-elevated Akt activity. Given that NGF exerts trophic effects via TrkA binding, it was expected that L5/LOX-1 would interfere with NGF-induced neuronal differentiation by disrupting TrkA. TrkA phosphorylation at specific sites is required for activation of various kinase pathways (Tyr490 for Shc/Ras/MAPK, Tyr751 for PI3K/Akt, Tyr785 for PLCγ/PKC/MAPK) and elevated TrkA kinase activity (Tyr674/675) [29]. However, L5 had no effect on overall phosphotyrosine status after NGF stimulation or on NGF-induced ERK, JNK, and p38 activation. This illustrates that L5/LOX-1 selectively weakens intracellular PI3K/Akt signaling, but not TrkA receptor activity or downstream MAPK pathways. Our findings agree with previous evidence indicating that L5 executes its biological effects via PI3K/Akt. For example, L5 activates CXCR2/PI3K/NFκB signaling, indirectly inducing cardiomyocyte apoptosis [57]. In ECs and endothelial progenitor cells as well, the PI3k/Akt pathway is inhibited by L5/LOX-1 [15,58]. Although our findings indicate that L5/LOX-1 suppresses TrkA/PI3K/Akt-associated neuronal differentiation in PC12 cells, more detailed mechanisms are still unclear at the present stage. Profound studies are needed in the future.

**Figure 9:**
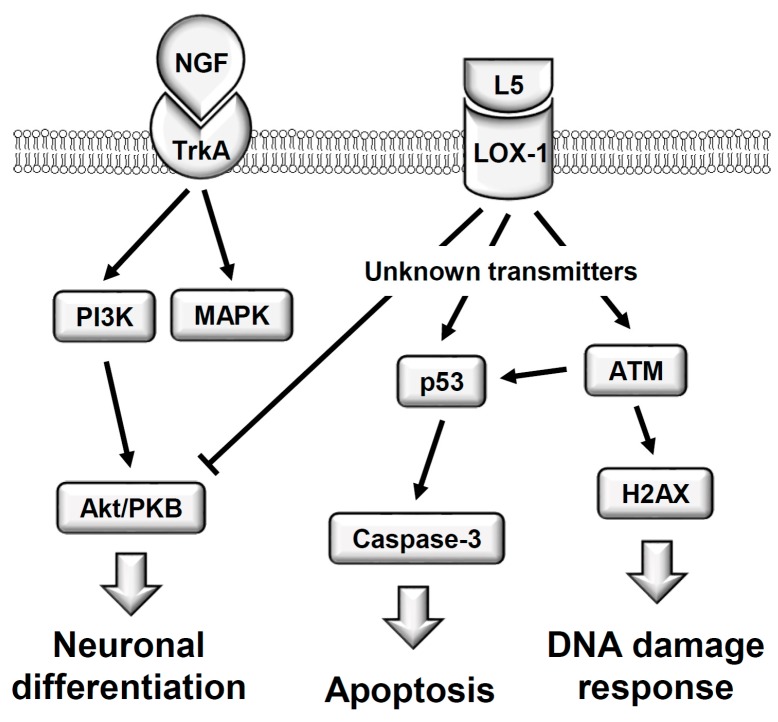
A schematic illustration shows the L5-induced cytotoxicity in PC12 cells. Through LOX-1 and unknown transmitters, L5 particle triggers ATM/H2AX-associated DNA damage response, activates p53/caspase 3-dependent apoptosis, and impairs NGF-induced neuronal differentiation (i.e., the suppression of NGF-activated PI3K/Akt cascade).

In summary, our findings reveal, for the first time, the direct cytotoxicity of LDL(−), especially fraction L5, to neuron-like PC12 cells. We demonstrate that L5 concentration- and time-dependently reduces cell viability by inducing ATM/H2AX-associated DNA damage and LOX-1/p53/caspase-3-dependent apoptosis; moreover, sublytic concentration of L5 inhibits NGF-induced neuronal differentiation by LOX-1-mediated suppression of the PI3K/Akt cascade (Figure 9). High circulating LDL is strongly implicated in the pathophysiology of neurodegenerative disorders such as AD-type dementia. Therefore, lowering plasma LDL(−) and inhibiting electronegative modification of LDL should be considered primary prevention measures for cognitive impairment and AD.

## 4. Materials and Methods

### 4.1. Chemicals, Antibodies, Kits and Reagents

All chemicals were from Sigma-Aldrich (Saint Louis, MO, USA) unless stated otherwise. Comet assay kits, reagents and slides were purchased from Trevigen (Gaithersburg, MD, USA). Antibodies, including phospho-ERK (pERK), pJNK, pp38, pAkt, pp53, pTrkA, ERK, JNK, p38, p53, ATM, H2AX, Egr-1, NF-M, TrkA, and cleaved caspase-3 were purchased from Cell Signaling Technology (Beverly, MA, USA). The pATM and γH2AX antibodies were obtained from Millipore (Billerica, MA, USA). Akt, LOX-1 and GAPDH antibodies, and siRNA were obtained from Santa Cruz Biotechnology (Santa Cruz, CA, USA). Alexa Fluor 488-conjugated antibody and Lipofectamine 2000 reagent were purchased from ThermoFisher Scientific (Waltham, MA, USA). Recombinant human β-NGF and caspase inhibitor Z-VAD-FMK were purchased from R & D System (Minneapolis, MN, USA). Horseradish peroxidase-conjugated anti-mouse and anti-rabbit IgG antibodies were purchased from Jackson ImmunoResearch Laboratories (West Grove, PA, USA). All cell culture reagents were obtained from Gibco-BRL/Invitrogen (Carlsbad, CA, USA).

### 4.2. Obtainment of Human Plasma LDL

Plasma LDL was obtained from blood samples of hypercholesterolemic patients (diagnosed by physicians) in Kaohsiung Medical University (KMU) Hospital. All participants provided written consent, and ethics committees (Institutional Review Board of KMU Hospital) reviewed and approved the consent procedure. The preparation of LDL particles was at Lipid Science and Aging Research Center (LSARC) of KMU, Kaohsiung, Taiwan. Initially, LDL isolation was done by sequential potassium bromide density centrifugation. The yielding LDL at a density of 1.019 to 1.063 g/mL was treated with 5 mM EDTA and nitrogen to avoid ex vivo oxidation. After removing salts by dialysis (20 mM Tris-HCl [pH 8.0], 0.5 mM EDTA and 0.01% NaN_3_), LDL was further separated into subfractions of L1 to L5 according to electrical charge by using a fast protein liquid chromatography with a UnoQ12 column (Bio-Rad Laboratories, Inc., Hercules, CA, USA), as described previously [18,59]. OxLDL was prepared by incubating L5-free LDL (L1) with 5 μM CuSO_4_ at 37 °C for 24 h. All LDL particles were sterilized by passing through 0.22 μm filters before using for cell experiments.

### 4.3. Cell Culture

Rat PC12 cells (American Type Culture Collection, Rockville, MD, USA) were grown as monolayer cultures and maintained in Dulbecco’s modified Eagle’s medium (DMEM) containing 10% horse serum, 5% heat-inactivated fetal bovine serum, and 1% penicillin/streptomycin. In order to induce morphological differentiation, cells (5 × 10^4^ cells/mL) were first subcultured in multiwell culture plates previously coated with poly-l-lysine (0.1 mg/mL). One day later, cells were rinsed once with fresh DMEM, followed by NGF (50 ng/mL) treatment for the indicated time periods in low serum medium composed of DMEM, 1% horse serum, and 1% penicillin/streptomycin. The medium containing NGF was replaced every 2 days when long-term treatment was executed.

### 4.4. MTT Reduction Assay

For the determination of cell viability, a biochemical method based on 3-(4, 5-dimethylthianol-2-yl)-2, 5-diphenyltetrazolium bromide (MTT) reduction was used to assay the metabolic activity of cultured cells. Stock MTT solution (5 mg/mL) was added to all cell-containing wells and diluted to a final concentration of 0.5 mg/mL. After incubation at 37 °C for 2 h, the MTT-containing cell medium was removed. The purple formazan crystals yielded by the action of mitochondrial dehydrogenase was dissolved with dimethyl sulfoxide and quantified spectrophotometrically. The light absorbance values at 570 nm against a 630 nm reference wavelength (ΔOD; OD: optical density) were recorded. The cell survival percentage is calculated by the formula: (viable cells) % = (ΔOD of treated sample/ΔOD of untreated control) × 100. The untreated control well was assigned a percentage value of 100% cell survival.

### 4.5. siRNA Transfection

Cells were cultured in growth medium without antibiotics for 24 h prior to transfection. According to the manufacturer’s protocol, transfection of siRNA duplex (100 pmol) in 6-well culture plates was performed using Lipofectamine 2000 reagent and Opti-MEM. After maintaining transfected cells for 6 h, the culture medium was replaced with fresh growth medium. Western blotting was used for determining the knockdown effect of siRNA on target protein. All siRNA oligos were designed and synthesized by Santa Cruz Biotechnology. Rat LOX-1 siRNA (sc-156076) is a pool of 3 target-specific 19–25 nt siRNAs. The specific sequences are as follows: (i) LOX-1 siRNA-1 sense, 5′-CUGUAAGCUACUACAUAGATT-3′ and antisense, 5′-UCUAUGUAGUAGCUUACAGTT-3′; (ii) LOX-1 siRNA-2 sense, 5’-CCAUUAUGCUAGAGGUAAUTT-3′ and antisense, 5′-AUUACCUCUAGCAUAAUGGTT-3′; (iii) LOX-1 siRNA-3 sense, 5′-CUAGAACAAACACCAAUCUTT-3′ and antisense, 5′-AGAUUGGUGUUUGUUCUAGTT-3′. Control siRNA (sc-37007) is a non-targeting 20–25 nt siRNA designed as a negative control and leads to no degradation of any known cellular mRNA. The manufacturer does not offer the sequence of scrambled control siRNA.

### 4.6. Comet Assay

A neutral comet assay (single cell gel electrophoresis assay) was used to detect DSBs, as per the manufacturer’s instructions. After finishing electrophoresis, at least 50 randomly selected images were analyzed from each treatment group under a fluorescence microscope (Nikon ECLIPSE Ts2 microscope, Nikon, Inc., Melville, NY, USA) equipped with a fluorescein isothiocyanate filter set. Images were captured using a cooled CCD camera (Spot, Diagnostic Instruments, Sterling Heights, MI, USA). As an informative and reliable DNA damage parameter, the Olive tail moment, which is defined as the product of the amount of DNA in the tail (fraction of total DNA in the comet tail) and the mean distance of migration in the tail (comet tail length), was calculated. The quantifications of the comet tail length and olive tail moment were conducted using the COMETscore.v1.5 image processing software.

### 4.7. Immunofluorescence Detection of DNA DSBs

Cultures grown on coverslips were subjected to immunostaining. To detect DNA DSBs, cells were fixed in 4% paraformaldehyde, permeabilized with 0.3% Triton X-100 in phosphate buffered saline, and then incubated with 5% normal goat serum to block nonspecific binding. Subsequently, an antibody against phosphorylated H2AX (γH2AX, 1:200 dilution) was used overnight at 4 °C, followed by the corresponding secondary antibody conjugated to Alexa Fluor 488. The nuclei were observable by labelling with 4’, 6-diamidino-2-phenylindole (DAPI). After rinsing the labelled cells with phosphate buffered saline and mounting slides with an anti-fading aqueous medium, observation was carried out under a fluorescent microscope equipped with appropriate filters and a digital camera.

### 4.8. Western Blotting Analysis

Cells were collected and homogenized in ice-cold RIPA buffer as described previously [59]. After a 20-min incubation at 4 °C, cell extracts were centrifuged at 14,000× *g* for 10 min and protein concentration in the supernatant was determined with a Bio-Rad protein assay (Bio-Rad, Hercules, CA, USA). Cell lysates (40 μg/lane) were separated by electrophoresis through 8–12% SDS-polyacrylamide gels and then electroblotted to 0.45 μm PVDF membranes (Millipore, Bedford, MA, USA) using a semi-dry transfer apparatus (Hoefer Scientific Instruments, San Francisco, CA, USA). Membranes were blocked in 5% non-fat dry skim milk for 1 h at room temperature, followed by immunoblotting for desired proteins with the assist of specific primary a ntibodies and appropriate HRP-conjugated secondary antibodies. Protein bands developed on the X-ray film were visualized by an enhanced chemiluminescence kit (Amersham Biosciences, Piscataway, NJ, USA). Analyses of protein band densities were performed using an Image J software (NIH, Bethesda, MD, USA).

### 4.9. Statistical Analysis

Treatment group means were compared by ANOVA, followed by Dunnett’s test or Bonferroni’s *t*-test for pair-wise comparisons. Paired groups were compared by Student’s *t*-test. All results are expressed as the mean ± standard error of the mean (SEM). Differences were considered significant when the probability value was less than 0.05 (*p* < 0.05). SigmaStat version 4.0 software (Jandel Scientific, San Diego, CA, USA) was used for all statistical analyses.

## Abbreviations

AD	Alzheimer’s disease
ApoB	Apolipoprotein B
ATM	Ataxia-telangiectasia mutated
BBB	Blood-brain barrier
CSF	Cerebrospinal fluid
CVD	Cardiovascular disease
DAPI	4′,6-Diamidino-2-phenylindole
DMEM	Dulbecco’s modified Eagle’s medium
DSBs	Double strand breaks
EC	Endothelial cell
Egr-1	Early growth response-1
ERK	Extracellular signal-regulated kinase
JNK	c-JUN NH2-terminal protein kinase
LDL	Low-density lipoprotein
LDL(−)	Electronegative low-density lipoprotein
LDL-C	LDL-cholesterol
LDLR	LDL receptor
LOX-1	Lectin-like oxidized low-density lipoprotein receptor-1
MAPKs	Mitogen-activated protein kinases
MTT	3-(4,5-Dimethylthianol-2-yl)-2,5 diphenyltetrazolium bromide
NGF	Nerve growth factor
NF-M	Neurofilament-medium
OxLDL	Oxidized LDL
PFT-α	Pifithrin-α
